# A new twist in measuring mutation rates

**DOI:** 10.7554/eLife.29586

**Published:** 2017-07-14

**Authors:** Bartram L Smith, Claus O Wilke

**Affiliations:** Department of Integrative Biology, The University of Texas at Austin, Austin, United States; Department of Integrative Biology, The University of Texas at Austin, Austin, United Stateswilke@austin.utexas.edu

**Keywords:** mutation rate, diversity, evolution, Virus

## Abstract

The influenza virus mutates faster than we previously thought.

**Related research article** Pauly M, Procario M, Lauring A. 2017. A novel twelve class fluctuation test reveals higher than expected mutation rates for influenza A viruses. *eLife* **6**:e26437. doi: 10.7554/eLife.26437

Every year the World Health Organization Influenza Surveillance Network reviews staggering amounts of data to help predict which strains of influenza virus will be suitable candidates for a flu vaccine for the coming influenza season (Barr et al., 2010). This review is necessary because of the appearance in most years of new mutant strains that can bypass the immunity provided by the previous year's vaccine. The rapid turnover of the different strains of the virus circulating in human populations is largely due to the virus being able to rapidly accumulate new mutations (Duffy et al., 2008).

The frequency with which new mutations occur (known as the mutation rate) influences the ability of a virus to adapt and evade the host’s immune system, and researchers have long been interested in accurately measuring these mutation rates (Parvin et al., 1986; Nobusawa and Sato, 2006). However, existing approaches to measuring mutation rates may have potential biases and shortcomings that have not been fully explored or corrected for. Now, in eLife, Matthew Pauly, Megan Procario and Adam Lauring of the University of Michigan report that using a new twist on an old method can overcome the major flaws of a current approach (Pauly et al., 2017).

When an influenza virus infects a host cell it tricks the cell into copying its genome (which is encoded in RNA rather than DNA) and assembling new virus particles, known as progeny virions. A viral enzyme known as RNA polymerase works with molecular machinery in the host cell to copy the viral RNA. However, this enzyme frequently makes mistakes, leading to a high rate of mutations in the new RNA molecules. Alongside this process, the cell uses sections of the viral RNA (called transcripts) as templates to make the proteins that are the building blocks of the progeny virions.

A widely used method of measuring mutation rates involves sequencing the genome of a virus, then allowing the virus to infect cells, sequencing the genomes of the progeny virions and, lastly, comparing the original genome sequence and the progeny sequences in order to identify the mutations that have arisen during the infection cycle (Sanjuán et al., 2010). This sequencing approach has the advantage that it provides both a total count of mutations and the frequencies of the different types of mutations (such as A to U, C to G, and so on). However, there are two potential problems with this method. First, it can be difficult to distinguish genuine mutations from errors introduced during sequencing. Second, the sequencing approach may be missing important mutations. For example, mutations that crop up early in the infection cycle may reduce the virus’s ability to replicate, thus biasing the resulting progeny virions away from those mutations.

Pauly et al. were able to sidestep the first problem by also sequencing transcripts from an artificial DNA construct known as a plasmid that is based on the RNA encoding some of the virus genome. Both the plasmid sequences and the viral genome sequences are expected to experience similar amounts of sequencing errors, so any difference in the observed mutation frequencies must be caused by mistakes made by the viral polymerase as it copied the viral genome. This technique revealed that sequencing errors account for at least half of the mutations found in the influenza virus using the standard sequencing approach.

To assess the severity of the second problem, Pauly et al. looked at the number of mutations that result in the production of incomplete proteins, which are generally lethal to the virus. They found that the viral genomes experienced many fewer mutations of this type than the plasmid sequences (which were not under any selective pressure). Thus, it appears that lethal or very harmful mutations can be missed in the sequencing-based approach to measuring mutation rates.

As an alternative to sequencing viral genomes, it is also possible to measure mutation rates using a fluctuation test. This approach – which was first developed by Max Delbrück and Salvador Luria in the early 1940s (Luria and Delbrück, 1943) – relies on counting rare mutations to an easily observable phenotype, such as resistance to a drug. The main limitation of the traditional fluctuation test is that it cannot directly measure the rates at which individual nucleotides within RNA or DNA are changed by mutations. However, this limitation could be overcome if it were possible to pin-point exactly which mutations cause the measured phenotype.

This is exactly what Pauly et al. did: they developed a fluctuation test for influenza virus based on the fluorescence emitted by green fluorescent protein (GFP). This involved producing recombinant influenza viruses that expressed a version of GFP with a single-nucleotide change that removed the fluorescent properties of the protein. Mutations that reverse this change restore fluorescence, making it possible to count how often such a reversion mutation occurs (Figure 1). Importantly, Pauly et al. were able to construct 12 different recombinant viruses that required 12 different single-nucleotide reversion mutations to restore fluorescence, one for each possible mutation class. These mutant GFPs do not alter the ability of the viruses to infect cells and replicate, so these fluctuation tests are expected to be free from the problem of lethal mutations seen in the sequencing-based approach.

**Figure 1.**
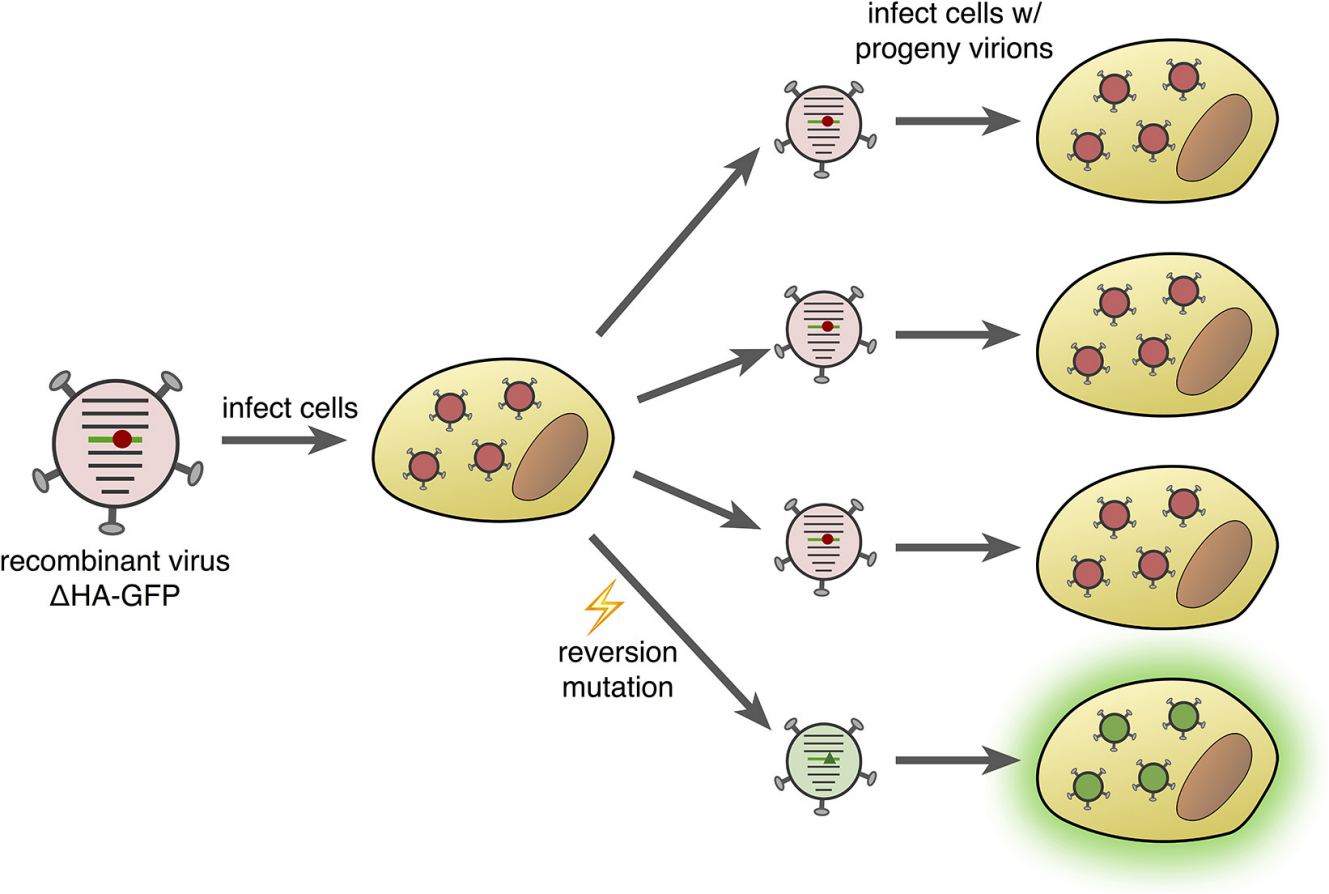
Fluorescence-reversion fluctuation test for the influenza virus. Pauly et al. started with a recombinant influenza strain known as ΔHA-GFP in which the gene encoding the hemagglutinin surface protein (HA) had been replaced by a gene encoding a version of green fluorescent protein (GFP). This GFP gene contained a single point mutation (shown as a red circle) that prevented the protein from producing green fluorescence. The ΔHA-GFP viruses were allowed to infect mammalian cells and replicate. If, during the first round of replication, a reversion mutation occurs at the site of the original mutation (green triangle), then green fluorescence is restored to GFP. If this particular virus particle then infects a mammalian cell, its progeny virions also produce green fluorescence (bottom right). The ratio of fluorescing to non-fluorescing infected cells in the second round of infection provides an estimate of the mutation rate for this specific reversion mutation.

Using their new test, Pauly et al. found that the rate at which the influenza virus mutates may be more than double the rates that had been previously reported. This information will undoubtedly help in developing better models of influenza evolution, potentially allowing for better predictions of the changes in circulating strains that allow the viruses to bypass existing vaccines. More importantly, the method has applications beyond just the influenza virus, as it should work with any virus that can tolerate the gene encoding GFP being inserted into its genome. Accurate measurements of mutation rates for other viruses with RNA genomes could be valuable in numerous ways, from assisting in the development of new vaccines (Ojosnegros and Beerenwinkel, 2010) to informing the development of treatments that disable viruses by inducing harmful mutations (Bull et al., 2007).

## References

[bib1] Barr IG, McCauley J, Cox N, Daniels R, Engelhardt OG, Fukuda K, Grohmann G, Hay A, Kelso A, Klimov A, Odagiri T, Smith D, Russell C, Tashiro M, Webby R, Wood J, Ye Z, Zhang W, Writing Committee of the World Health Organization Consultation on Northern Hemisphere Influenza Vaccine Composition for 2009-2010 (2010). Epidemiological, antigenic and genetic characteristics of seasonal influenza A(H1N1), A(H3N2) and B influenza viruses: basis for the WHO recommendation on the composition of influenza vaccines for use in the 2009-2010 northern hemisphere season. Vaccine.

[bib2] Bull JJ, Sanjuán R, Wilke CO (2007). Theory of lethal mutagenesis for viruses. Journal of Virology.

[bib3] Duffy S, Shackelton LA, Holmes EC (2008). Rates of evolutionary change in viruses: patterns and determinants. Nature Reviews Genetics.

[bib4] Luria SE, Delbrück M (1943). Mutations of bacteria from virus sensitivity to virus resistance. Genetics.

[bib5] Nobusawa E, Sato K (2006). Comparison of the mutation rates of human influenza A and B viruses. Journal of Virology.

[bib6] Ojosnegros S, Beerenwinkel N (2010). Models of RNA virus evolution and their roles in vaccine design. Immunome Research.

[bib7] Parvin JD, Moscona A, Pan WT, Leider JM, Palese P (1986). Measurement of the mutation rates of animal viruses: influenza A virus and poliovirus type 1. Journal of Virology.

[bib8] Pauly MD, Procario MC, Lauring AS (2017). A novel twelve class fluctuation test reveals higher than expected mutation rates for influenza A viruses. eLife.

[bib9] Sanjuán R, Nebot MR, Chirico N, Mansky LM, Belshaw R (2010). Viral mutation rates. Journal of Virology.

